# Δ-FeOOH as Support for Immobilization Peroxidase: Optimization via a Chemometric Approach

**DOI:** 10.3390/molecules25020259

**Published:** 2020-01-08

**Authors:** Tássia Silva Tavares, Eduardo Pereira da Rocha, Francisco Guilherme Esteves Nogueira, Juliana Arriel Torres, Maria Cristina Silva, Kamil Kuca, Teodorico C. Ramalho

**Affiliations:** 1 Department of Chemistry, Federal University of Lavras, N° 37, Lavras, MG 37200-000, Brazil; tassiastavares@hotmail.com (T.S.T.); eduardo.rocha@ifsudestemg.edu.br (E.P.d.R.); julianaarriel@hotmail.com (J.A.T.); crisiria@ufsj.edu.br (M.C.S.); 2 Department of Chemistry Engineering, Federal University of São Carlos, 13565-905 São Carlos, SP, Brazil; nogueira@ufscar.br; 3 Department of Chemistry, Faculty of Science, University of Hradec Králové, 500 03 Hradec Králové, Czech Republic

**Keywords:** immobilization, bioremediation, horseradish peroxidase, iron oxide hydroxide

## Abstract

Owing to their high surface area, stability, and functional groups on the surface, iron oxide hydroxide nanoparticles have attracted attention as enzymatic support. In this work, a chemometric approach was performed, aiming at the optimization of the horseradish peroxidase (HRP) immobilization process on Δ-FeOOH nanoparticles (NPs). The enzyme/NPs ratio (X1), pH (X2), temperature (X3), and time (X4) were the independent variables analyzed, and immobilized enzyme activity was the response variable (Y). The effects of the factors were studied using a factorial design at two levels (−1 and 1). The biocatalyst obtained was evaluated for the ferulic acid (FA) removal, a pollutant model. The materials were characterized by X-ray powder diffraction (XRD), Fourier transform infrared spectroscopy (FTIR), and scanning electron microscopy (SEM). The SEM images indicated changes in material morphology. The independent variables X1 (−0.57), X2 (0.71), and X4 (0.42) presented the significance effects estimate. The variable combinations resulted in two significance effects estimates, X1*X2 (−0.57) and X2*X4 (0.39). The immobilized HRP by optimized conditions (X1 = 1/63 (enzyme/NPs ratio, X2 = pH 8, X4 = 60 °C, and 30 min) showed high efficiency for FA oxidation (82%).

## 1. Introduction

Chemistry can be considered a fundamental science for sustainable development, possible processes that lead to the production of less waste, reducing the generation of toxic effluents. Nowadays, the application of biodegradable catalysts such as enzymes is highlighted in pollutant bioremediation and associated directly with the concept of Green Chemistry [1,2,3,4].

Peroxidases are oxidoreductases able to oxidize a wide variety of organic pollutants of environmental importance. Horseradish peroxidase has a versatile application, and it is highlighted in the wastewater remediation, containing dyes, amines, and phenolic compounds [5,6,7,8,9]. This enzyme catalyzes the conversion of organic compounds in the presence of hydrogen peroxide, producing phenoxy radicals. These radicals react with each other, forming water-insoluble polymers, possible to separate by sedimentation [5,10]. However, soluble enzymes recovery is a limitation in the enzymatic process. Thereby, the facilitated separation of enzymes becomes an issue indispensable for advantageous processes, what can be reached by the enzymatic immobilization.

Enzyme immobilization is an attractive technique to achieve reuse and stabilization of biocatalysts [11,12,13,14]. Different methods have investigated the enzymatic immobilization, being the adsorption frequently exploited [13,15,16], which involves covalent bonds embracing physical, chemical, and electrostatic interactions. Immobilization processes by covalent bonds are highlighted owing to the possibility of obtaining stable catalysts [17]. In this case, the immobilization processes may occur in two ways: randomly or oriented. Random processes based on whether interactions between the amino acid lysine (Lys) and terminal amino acid and the support occur without the addition of functional groups on the support surface conducting the no-oriented immobilization processes. In contrast, the addition of functional groups leads to reaction with the amino group residues of the protein directly. If the functionalization of the support is unnecessary, the process can be simplified. However, it occurs when the support has functional groups where the enzymes bind [13,18].

In this context, iron oxides nanoparticles stand out as a potential molecule support [14,19]. Iron hydroxyl oxide (FeOOH) has an attractive surface with the presence of hydroxyl groups (OH-), which may facilitate enzyme attachment. Besides, it exhibits unique characteristics (e.g., high surface area, magnetism, no-toxicity, and excellent stability) [15,20,21] and overcomes drawbacks regarding functionalization steps. Accordingly, the iron hydroxyl oxide (Δ-FeOOH) presents an excellent proposal as molecule immobilization, as the surface interaction of the enzyme is possible without requiring the addition of functional groups. Compared with other methods of enzyme immobilization technologies reported previously [15,16], this material as an enzymatic support can reduce the costs of the process, resulting in a low-cost magnetic nanocatalyst obtained.

To the best of the authors’ knowledge, the study of iron hydroxyl oxide not functionalized (Δ-FeOOH) as a support has not yet been reported. On the other hand, obtaining a catalytically efficient nanobiocatalyst depends the processing technique. Therefore, this work investigated horseradish peroxidase (HRP) immobilization on Δ-FeOOH nanoparticles, approaching a systematic chemometric study aiming to optimize the immobilization process. A response surface methodology (RSM) was applied by adopting a statistical design of experiments (SDEx) at two levels (−1 and 1). The process performed by SDEx was recognized as an efficient tool to optimize chemical processes [22], improving the conditions minimizing the experiment number [22,23]. Associated with the “Green Chemistry” principles, this approach minimizes the amount of the reagents in the process optimization, generating a smaller quantity of chemical residues.

The optimization development served for the further study of immobilization parameters such as support amount, pH, reaction time, and temperature. Characterization studies performed using X-ray diffraction (XRD), Fourier transform infrared spectroscopy (FTIR), and Scanning electron microscopy (SEM) evaluated the particles synthesized. Oxidation of ferulic acid (a lignin model compound) assessed the performance of the catalyst obtained.

## 2. Materials and Methods

### 2.1. Chemical Reagents

Ferulic acid (FA) and horseradish peroxidase (EC 1.11.1.7 ≥ 146 units per mg protein), as well as the other reagents, were obtained from Sigma-Aldrich (St. Louis, MO, USA).

### 2.2. Δ-FeOOH Nanoparticles’ Synthesis

The Δ-FeOOH nanoparticles were synthesized according to the modified procedure described by Tavares and collaborators [15]. Typically, 31.36 g of Fe(NH_4_)_2_(SO_4_)_2_·6H_2_O was dispersed into 200 mL of deionized water. Then, 100 mL solution of NaOH (5 M) was immediately added under continuous shaking. Then, 20 mL of 50% H_2_O_2_ was injected into it to provide rapid oxidation under constant stirring for 30 min. After the formation of Δ-FeOOH particles, the supernatant was filtered through a filter under vacuum and washed three times with deionized water. Finally, the particles were dried in an oven for 12 h at 60 °C.

### 2.3. Characterization of Materials

X-ray powder diffraction (XRD) was used to identify the crystal structure and to determine the average particle size. A Shimadzu XRD-6000 system with Cu Kα radiation (λ = 0.15406 nm) was used, with the scanning step of 0.02, count time of 6 s, and 2θ range from 10 to 80. The Scherrer equation was used to determine the average particle size, after correcting for instrumental broadening effects. From the scanning electron microscopy (SEM) (LEO 440 with an Oxford detector with an electron beam operating at 15 kV), the material’s morphology was determined. The materials’ surface groups were determined by Fourier transform infrared spectrophotometry (FTIR Spectrometer, PerkinElmer Spectrum 2000 Spectrometer) with spectral range from 400 to 4000 cm^−1^.

### 2.4. HRP Immobilization

The enzymatic solution of HRP (0.8 mg/mL; pH 4.0 citrate-phosphate buffer, and pH 8.0 Tris-HCl buffer 0.05 molL^−1^) was added to the nanoparticles and left at the desired temperature and time, under constant stirring. The suspensions were submitted to an external magnet for three min to separate the particles. Next, the particles were washed with buffer (3 mL) and distilled water (3 mL), and dried at 25 °C for 24 h. Measurements of activity were carried out from particles’ washing waters, intending to verify the possibility of the particles coming out from the support. Next, the biocatalyst was stored at 4 °C. Aliquots were taken at the end of each time to determine the residual enzyme activity and the maximum immobilization yield.

#### 2.4.1. Enzymatic Activity

The activity measurements were conducted according to the classical assay described by Khan and Robison [24]. The activity was expressed as units (U). One unit of peroxidase activity represents the oxidation of 1 μmol of guaiacol per minute. The assays were performed using 1.5 mL of guaiacol 1% (*v*/*v*), 0.4 mL of H_2_O_2_ 0.3% (*v*/*v*), and 1.2 mL of 0.05 molL^−1^ phosphate buffer pH 7.0. The enzymatic solution (0.1 mL) was added at room temperature, and for the immobilized enzyme (20 mg of particles) at 30 °C. The assay described is based on the rate of guaiacol oxidation for the formation of tetraguaiacol (ε = 26.6 mM^−1^cm^−1^). Organic compounds’ oxidation by the peroxidase occurs from the catalytic cycle. Firstly, the native peroxidase [heme (Fe^3+^)] bind to the H_2_O_2_, resulting in a heterolytic cleavage between the oxygen atoms by electron transfer. A water molecule is released, forming an intermediary enzyme state (I), comprising an oxyferryl species (Fe^4+^ = O) and a radical porphyrin cation. In the next reaction step, the enzyme state (I) oxidizes the first organic compound molecule, releasing a radical product, and the enzyme state (II), an oxyferryl species (Fe^4+^ = O). Finally, the reactive enzyme state (II) is reduced by a second organic compound molecule, converting the enzyme back to the original form. Both enzyme states (I and II) are powerful oxidants [5].

#### 2.4.2. Process Optimization Strategy—HRP Immobilization

In this study, an experimental design was performed by Statistical Software^®^. Developing a convenient model using RSM enabled investigating the optimum performance parameters that influence the HRP immobilization. On the basis of four variables at two levels each, an experimental design for the quadratic model was obtained. The factorial design (a 2^4^ design) consisted of 16 sets runs, of which the four process variables chosen were pH, support loading, temperature, and immobilization time. The activity of the immobilized enzyme was defined as the response for this study. Factors were analyzed at low (−1) and high (1) levels, as shown in Table 1. The effects of the independent variables were evaluated at 95% significance.

The resulting model was examined by analysis of variance (ANOVA). The validity of the model was determined based on Fischer’s F-test (F-values), associated probability (*p*-values), as well as regression coefficient values and lack of fit test. The mathematical model developed by RSM was validated by performing three independents experiments. Also, the immobilization yield (IY) and immobilization efficiency (IE) of the process were calculated according to Equations (1) and (2) [13].

(1) $$\%\;IY=\frac{100\times \left(\mathrm{Immobilized}\;\mathrm{activity}\right)}{Starting\;activity}$$

(2) $$\%\;IE=\frac{100\times \left(\mathrm{Observed}\;\mathrm{activity}\left(\mathrm{particles}\right)\right)}{Immobilized\;activity}$$

### 2.5. Ferulic Acid Oxidation

The biocatalyst performance was evaluated in the FA removal. The magnetic biocatalyst (800 mg of particles, which corresponds to activity 1.48U), obtained from optimized immobilization condition, was dispersed into at a constant pH (citrate phosphate buffer pH 7.0) with the addition of 3.0 mL of FA solution (1 mM) and 0.8 mL of H_2_O_2_ (2 mM). The FA removal assays were carried out in glass flasks, and the mixture was kept under stirring for 30 min at 30 °C [15]. Two experimental controls were carried out, one in the absence of the enzyme and the other in the absence of hydrogen peroxide. The immobilized enzyme was removed from the medium by magnetic separation. Measurements of activity were carried out from supernatant, intending to verify the possibility of the particles coming out from the support. The FA residual concentration was measured by the Folin and Denis colorimetric method [25]. The removal percentage (%) was calculated from the difference between the initial and remaining concentration of FA after the reaction.

## 3. Results and Discussion

### 3.1. Characterization of Materials

**Figure 1:**
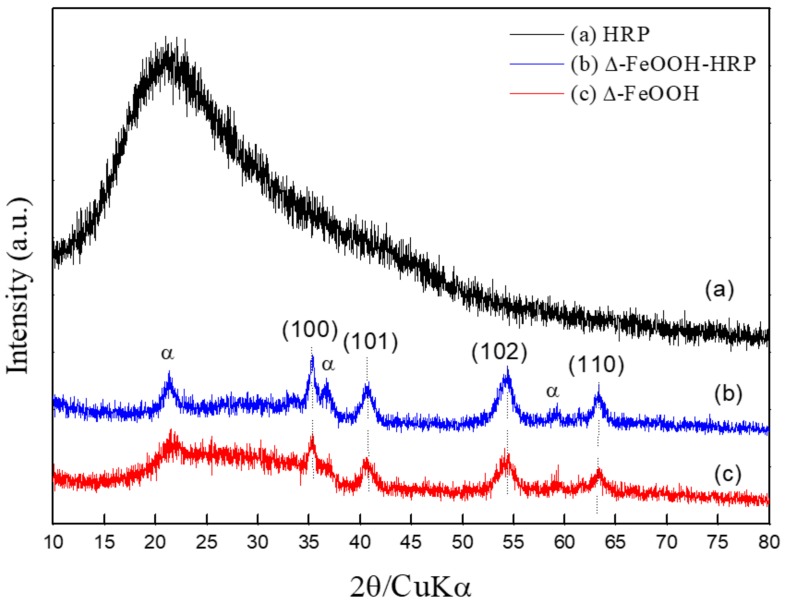
X-ray diffraction (XRD): (**a**) horseradish peroxidase (HRP); (**b**) Δ-FeOOH–HRP; (**c**) Δ-FeOOH particles.

The XRD results indicated that the particles of Δ-FeOOH were synthesized as a dominant phase. It presents the reflections of the planes (100) (101) (102) (110), which are consistent with the reported data at the International Centre for Diffraction Data (JCPDS N° 13-0087) for this oxide (Figure 1c). An amorphous halo displayed a single broad diffused peak centered on 2θ = 23°, which indicates the materials’ amorphous phase. Knowing that FeOOH has four different polymorphs denoted α, β, γ, and Δ, some peculiar characteristics such as chemical properties, composition, and nature mineralogical stand out among them. Commonly, the iron oxides mixed may occur as a result of the similar crystallization profile, and the octahedron hexagonal rings of Fe^+3^ possibly replace the isoforms during the formation of crystals, resulting different phases of FeOOH [26]. After the immobilization process, the goethite (α-FeOOH) phase was observed in the material (Figure 1b). Evidence of the α-FeOOH phase can indicate that a slight amount of Δ-FeOOH attached to the enzyme led to the replacement of isoforms.

**Figure 2:**
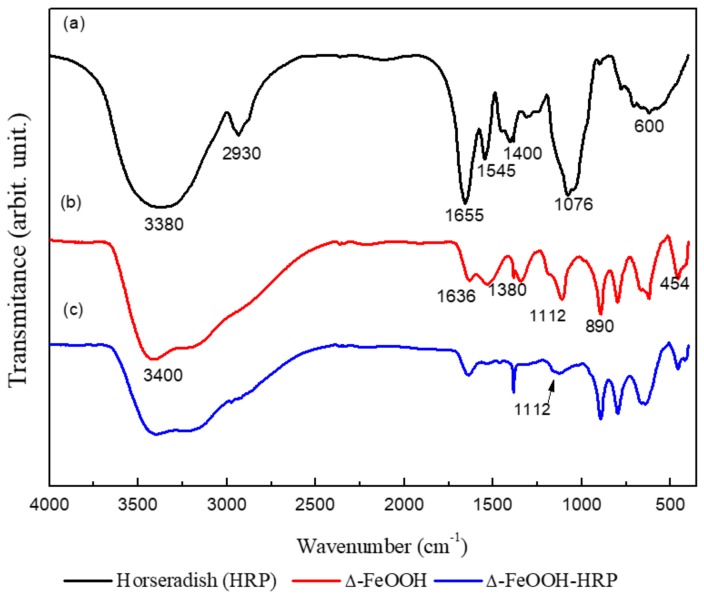
Fourier transform infrared spectroscopy (FTIR) spectra: (**a**) horseradish peroxidase (HRP); (**b**) Δ-FeOOH particles; and (**c**) Δ-FeOOH–HRP.

Regarding the chemical bonds on the surface of the particles, the materials’ characterization was also carried out by FTIR within the wavelength range of 500–4000 cm^−1^ (Figure 2). Observed spectral regions at 3380 cm^−1^ are attributed to O–H vibration modes, at 2930 cm^−1^ for the alkyl (-CH_2_) chains [27]. Peaks at 1655 and 1545 cm^−1^ assigned to the amide I (α-helix structure) [25] and 1400 cm^−1^ to several modes of NH (amide III) [28,29], while aliphatic amines (C–N stretching vibration) assign at the region 1076 cm^−1^. Around 600 cm^−1^, C–H bond deformation or bending for alkynes may be indicated. The single hydrogen of the terminal acetylene is characteristic at 680–610 cm^−1^, and OH of oxide hydroxide at 3400 cm^−1^. Bending vibrations of Fe–OH may be related to the peaks around at 1636 and 1112 cm^−1^, and the peaks at 890–454 cm^−1^ correspond to the metal–oxygen (Fe–O) [30]. However, vibrations of Fe–OH (1112 cm^−1^) are not displayed after immobilization, indicating a possible interaction between the enzyme and the support after the immobilization process.

**Figure 3:**
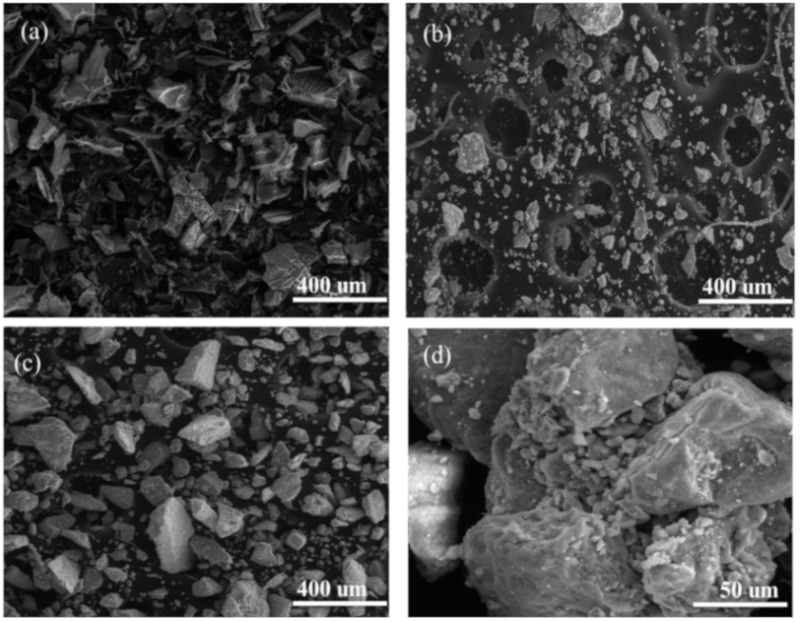
Scanning electron microscopy (SEM) image: (**a**) horseradish peroxidase (HRP); (**b**) Δ-FeOOH particles; and (**c**,**d**) Δ-FeOOH–HRP.

Scanning electron microscopy analyzed the morphological changes of the materials. The SEM images of HRP, pure Δ-FeOOH, and biocatalyst (Δ-FeOOH–HRP) are shown in Figure 3. The SEM micrographs (Figure 3b) show that the nanoparticles are structured in irregular compact blocks, and the enzyme is probably entrapped in these structures (Figure 3c,d).

### 3.2. Immobilization Process Optimization Strategy - HRP

To obtain the optimum immobilized enzyme activity, RSM was applied in this study. The obtained immobilized activity was analysed to develop a suitable regression model. A second-order quadratic regression surface model described the linear, quadratic, and interaction effects of four independent variables affecting the immobilization efficiency. The relationship between the experimental variables and response is fitted to a quadratic polynomial equation, as shown in Equation (3):

(3) Y=0.37−0.57X1+0.71X2−0.02X3+0.42X4−0.56X1 X2+0.03X1 X3−0.25X1 X4−0.035X2 X3+0.39X2 X4+0.11X3 X4.

**Figure 4:**
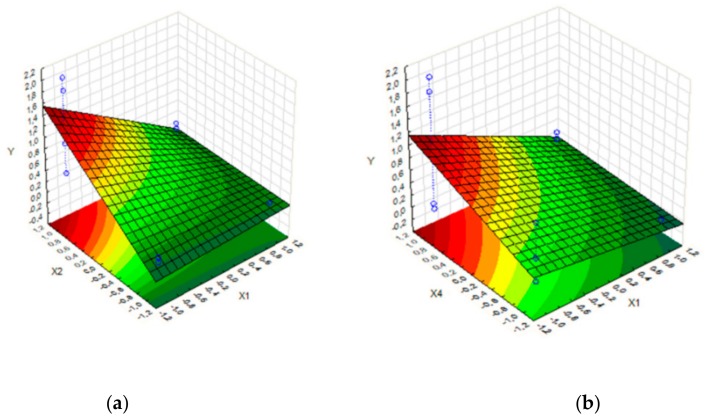
Response surface of factorial design: (**a**) variable X1 vs. X2; (**b**) variable X1 vs. X4.

The parameters NPs (X1), pH (X2), time (X3), and temperature (X4) are defined for the process optimization strategy. The effects of the independent variables were analyzed (Table 2). The independent variables that showed significant effects estimates were the variables X1 (−0.58), X2 (0.71), and X4 (0.42). In the combination of the variables, there were two significant effects estimates, X1*X2 (−0.57) and X2*X4 (0.39). The results showed that the change from the X1 level −1 (1/63 enzyme/NPs) to level 1 (1/313 enzyme/NPs) decreased 58% of immobilized HRP activity. Although the change in variable pH and temperature from level −1 (pH 4.0 and 25 °C) to level 1 (pH 8.0 and 60 °C) increased the activity in 71% and 42%, respectively, and in the combination of X2 (pH 8.0) and X4 (60 °C), the change caused a 39% increase in the activity. These effects can be observed in the surface response (Figure 4) for the changes’ interaction in the variables NPs and pH, and their influences under the enzymatic activity (Y), the dependent variable (further details are available in the Supplementary Materials).

Also, the response surface (Figure 4) showed the effects of the results influenced by the changes in variables X1 and X4. It could observed that, when the variable X1 is positive (1) and the variable X2 is positive (1), the results (Y) are lower. When the variable X1 is negative (−1) and the variable X2 is positive (1), the result assumed the higher value. Analyzing the response surface, it was possible to define the best immobilization parameters inside the experimental conditions. The optimized experiment indicated optimized conditions of X1 = 1/63 (enzyme/NPs), X2 = pH 8, and X4 = 60 °C (Figure 4, and Table 1: experiments 10 and 14). The variable X3 (time) does not change with the change to level −1 from 1. Consequently, its values were not significant. Therefore, 30 min was the time recommended.

**Figure 5:**
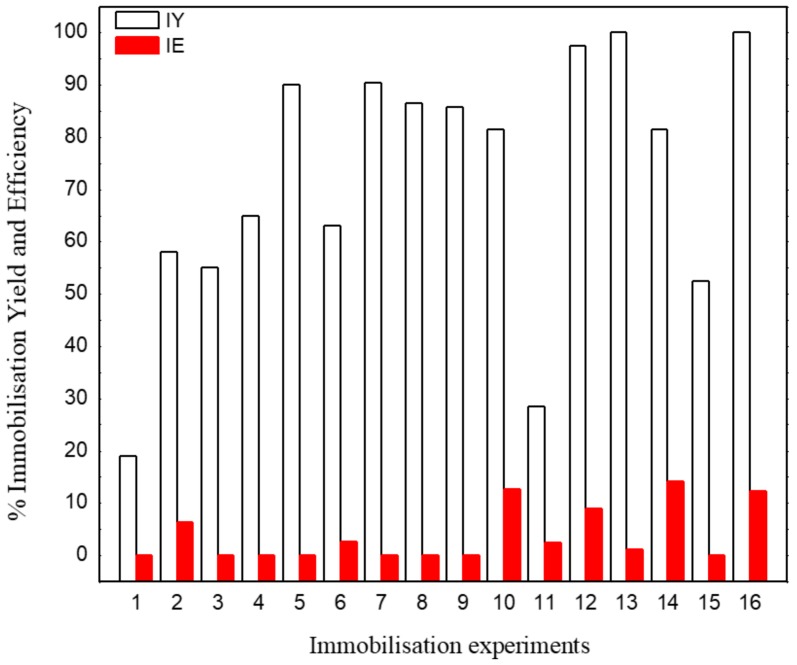
Immobilization yield (IY) and immobilization efficiency (IE): pH 4.0 (odd-number experiments), pH 8.0 (even-number experiments), and temperature 25 °C (experiments 1 until 8) and 60 °C (experiments 9 until 16).

**Figure 6:**
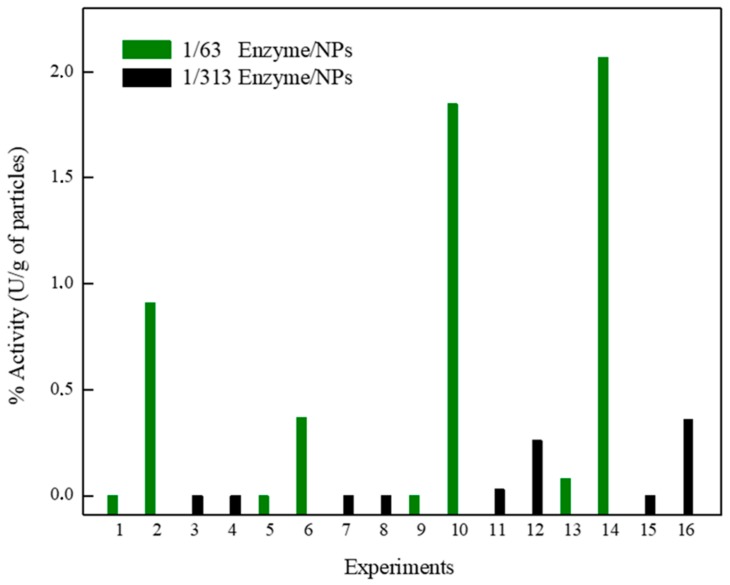
Activity (U/g of nanoparticles) of immobilized HRP on ferroxyte Δ-FeOOH under different processes conditions: pH 4.0 (odd-number experiments), pH 8.0 (even-number experiments), and temperature 25 °C (experiments 1 until 8) and 60 °C (experiments 9 until 16).

Assessing the immobilization yield, using a lower enzyme dose (1/313 enzyme/NPs at pH 8.0), the highest IY percentage (with the exception in pH 4.0 at 60 °C) was observed, indicating that the excess of enzymes may result in agglomerations on the surface of the support and affect the immobilization process. Already, processes conducted with pH 4.0, at 60 °C, and a lower enzyme dose (experiments 11 and 15) resulted in the lowest IY (29% and 52%, respectively) when compared with a more significant enzyme dose (experiments 9 and 13), which presented 86% and 100% of IY, respectively (Figure 5). However, the enzyme immobilized in these conditions (experiments 9 and 13) has not exhibited catalytically active (Figure 6). This is attributed to the inaccessible substrate to the surface or pores of the support with higher enzyme layers [11]. Also, the effect of the lowest pH can decrease the three-dimensional structure stability of the enzyme. HRP immobilized in the processes (2, 6, 10, and 14) involving the higher dose enzyme (1/63 enzyme/NPs) and pH 8.0 presented catalytic activity, displaying better activity when obtained at 60 °C than at 25 °C.

Nevertheless, under certain reaction conditions, the immobilization processes resulted in 0% of immobilization efficiency, or enzymatic activity was not found in the immobilized enzyme. It is possible to obtain 100% IY, and the lowest, or 0%, IY. This can occur when all the enzymes in solution are immobilized; however, no activity is found in the immobilized enzyme because the enzyme was deactivated or became inaccessible for some reason upon immobilization [13].

The immobilization at the highest temperature (60 °C) resulted in an excellent immobilization yield and the best activity of the immobilized HRP. The influence of temperature may lead to changes in the structure of the enzymes. Regardless of pH, the higher temperature can result in the HRP unfolding phase. Thus, the tertiary enzyme structure can be affected at around 45 °C, but without depletion of the heme group, which occurs at the highest temperature (74 °C) [31]. The interaction between HRP and support (Δ-FeOOH) was directly influenced by higher temperatures without compromising the activity after immobilization.

The immobilization pH may influence the charge state of the support’ surface. Considering the pHpzc 5.8 (pH at which the surface is zero charged) for the FeOOH [32], most of the hydroxyl groups are negatively charged when pH is above to pHpzc (Equations (4) and (5)). Otherwise, it predominates a surface positive charged when pH is below to pHpzc [32]. The alkaline pH may favor the HRP immobilization through amino groups positively charged interacting with hydroxyl groups negatively charged.

(4) $$Fe_{surface}^{3+}{\;}^{}-OH+H^{+}↔\;Fe_{surface}^{3+}{\;}^{}-\;OH_{2}^{+}\;\;\mathrm{pH}<\mathrm{pHpzc}$$

(5) $$Fe_{surface}^{3+}{}^{\;}-OH+OH^{-}↔Fe_{surface}^{3+}{\;}^{}-O^{-}+H_{2}O\;\;\mathrm{pH}>\mathrm{pHpzc}$$

The presence of hydroxyl groups, such as Fe–OH on the surfaces of the nanoparticles provides a versatile handle for the interaction of different functional groups. Different interactions can be involved in adsorption processes, which include electrostatic, hydrophobic, chelating, and covalent bonding. Hence, a synergistic effect of the combination of these interactions may favor the HRP immobilization on FeOOH nanoparticles. The immobilized horseradish peroxidase displayed 1.9 U/g, more significant activity than that reported for immobilized soybean peroxidase (0.87 U/g) on Δ-FeOOH modified [15], indicating that Δ-FeOOH, without the functionalization steps, is a promising support for the immobilization process. Although it is common in some cases, a specific enzyme amount comes out of the support after immobilization [11]; enzyme leaching from the support was not observed. Therefore, it was concluded that the interaction between the enzyme and Δ-FeOOH is strong enough to obtain a nanocatalyst.

### 3.3. Ferulic Acid Oxidation

The peroxidase showed a potential of 82% in the FA removal (800 mg of immobilized HRP—1.48U). The controls in the absence of peroxidase and hydrogen peroxide did not have oxidized FA oxidation, indicating that the oxidation occurred only by peroxidase immobilized. Thus, the FA oxidation shows that the bond between NPs and HRP does not block the enzymatic site where the FA has catalysed. Usually, the methoxy oxygen of FA is hydrogen-bonded to the arginine 38 residue at the substrate-binding site [33].

**Figure 7:**
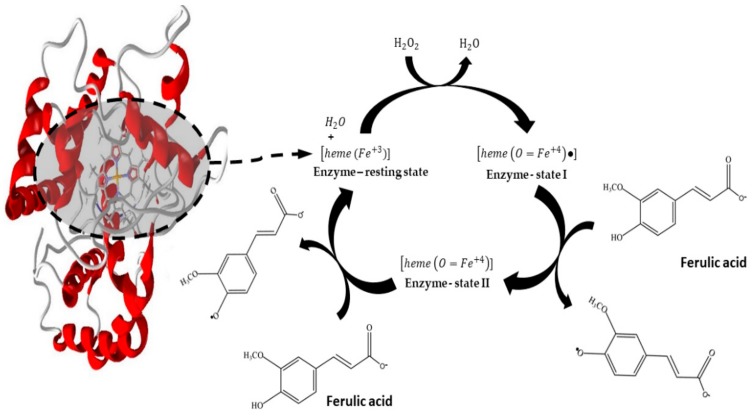
Peroxidases catalytic cycle involving the ferulic acid oxidation.

The peroxidase catalytic mechanism (Figure 7) responsible for FA oxidation includes the transfer of electrons. The first state of the modified enzyme (I) occurs from the reaction between H_2_O_2_ and the Fe^+3^ resting state, comprising Fe^+4^ and porphyrin radical cation. The enzyme stage (I) has two oxidation equivalents above the resting state. Then, the modified enzyme oxidizes the ferulic acid, resulting in another modified enzyme state (II), a Fe^+4^ no radical porphyrin cation. The ferulic acid oxidation in the two reduction steps leads to the radical species generation. These radicals are exceedingly reactive; therefore, the oxidized molecules product of this reaction reacts to each other, resulting in dimers, trimers, oligomers, and polymers [5,10,16], which can be separated by the simple process of sedimentation.

The immobilized enzymes’ conformation, as well as the diffusion of the substrate, seems to be favorable to the catalytic performance, as substantial removal of pollutants was achieved [12,14,34,35,36]. Overcoming the limitations of the free enzyme and peroxidase immobilized on Δ-FeOOH modified (Δ-FeOOH–SiO_2_–APTES–GLU) [15], this material is highlighted as an excellent support, even in the absence of functionalization steps. It can be assigned to its large area and functional groups on its surface, which interacts with the amino acid residues of the HRP without compromising catalytic efficiency.. Therefore, with the acquisition of an efficient nanocatalyst, from the support with attractive characteristics, and no requirement of functionalization steps, Δ-FeOOH becomes a support attractive for industrial applications.

## 4. Conclusions

In the present work, a magnetic biocatalyst was obtained via peroxidase immobilization onto the iron oxide. The enzyme successfully interacted with the ferroxyte nanoparticles, without the need to use silica coatings and the addition of functional groups. The optimized process allows for obtaining a reusable biocatalyst, in the absence of costly steps. Besides that, the biocatalyst showed excellent catalytic activity, which makes this process more interesting for industrial applications than free enzymes. Future studies will evaluate its performance in the removal of other organic pollutants. On the basis of the results, we conclude that HRP immobilized on ferroxyte can be considered a promising catalyst for environmental bioremediation.

## Figures and Tables

**Table 1:** Experiments matrix of 2^4^ fractional factorial.

Experiments	(X1) ^a^	(X2) ^b^	(X3) ^c^	(X4) ^d^
1	−1 (100)	−1 (4.0)	−1 (30)	−1 (25)
2	−1 (100)	1 (8.0)	−1 (30)	−1 (25)
3	1(500)	−1 (4.0)	−1 (30)	−1 (25)
4	1(500)	1 (8.0)	−1 (30)	−1 (25)
5	−1(100)	−1 (4.0)	1 (180)	−1 (25)
6	−1(100)	1 (8.0)	1 (180)	−1 (25)
7	1(500)	−1 (4.0)	1 (180)	−1 (25)
8	1(500)	1 (8.0)	1 (180)	−1 (25)
9	−1(100)	−1 (4.0)	−1 (30)	1 (60)
10	−1(100)	1 (8.0)	−1(30)	1 (60)
11	1(500) (1/313)	−1 (4.0)	−1 (30)	1 (60)
12	1(500)	1 (8.0)	−1 (30)	1 (60)
13	−1(100)	−1 (4.0)	1(180)	1 (60)
14	−1(100)	1 (8.0)	1 (180)	1 (60)
15	1(500)	−1 (4.0)	1 (180)	1 (60)
16	1(500)	−1 (8.0)	1 (180)	1 (60)

^a^ X1: nanoparticles (mg). ^b^ X2: pH. ^c^ X3: time (minutes). ^d^ X4: temperature (°C).

**Table 2:** Effects estimates from each independent variable of the factorial design 2^4^ evaluated at 95% of the coefficient limit.

Factor	Effects Estimate
Mean/Interc.	0.371250
X1	−0.578250
X2	0.712750
X3	−0.022750
X4	0.422000
X1*X2	−0.566000
X1*X3	0.038500
X1*X4	−0.257750
X2*X3	−0.035000
X2*X4	0.392250
X3*X4	0.113250

X1: enzyme/nanoparticles (NPs); X2: pH; X3: time (minutes); X4: temperature (°C).

## Supplementary Materials

The following are available online.
