# Supplementary material for: Δ-FeOOH as Support for Immobilization Peroxidase: Optimization via a Chemometric Approach
Source: Molecules. 2020 Jan 8;25(2):259. doi: 10.3390/molecules25020259 (PMC7024332; doi:10.3390/molecules25020259)
Supplement: Supplementary file 1 [file molecules-25-00259-s001.pdf]

Supplementary Materials

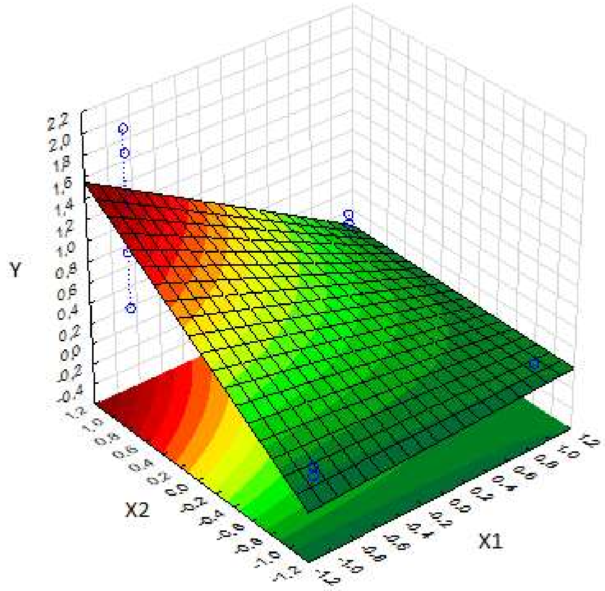

Figure S1 Response Surface of Factorial Design between Variable X1 vs X2

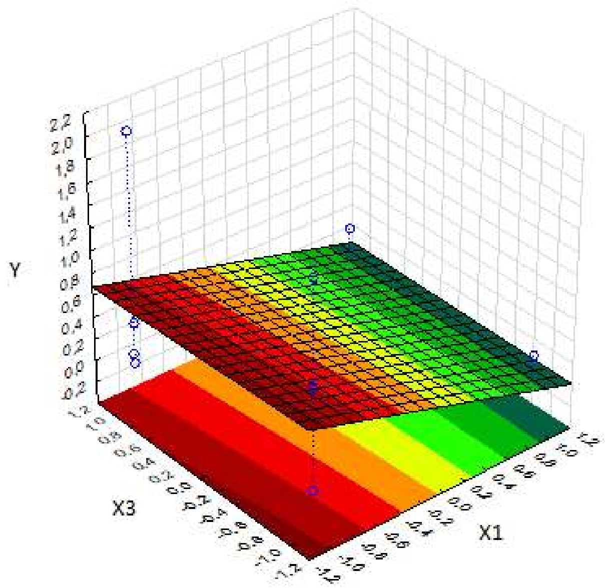

Figure S2 Response Surface of Factorial Design between Variable X1 vs X3

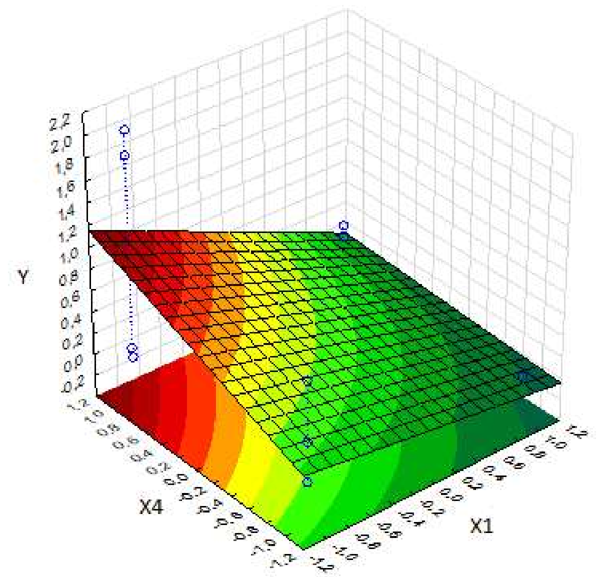

Figure S3 Response Surface of Factorial Design between Variable X1 vs X4

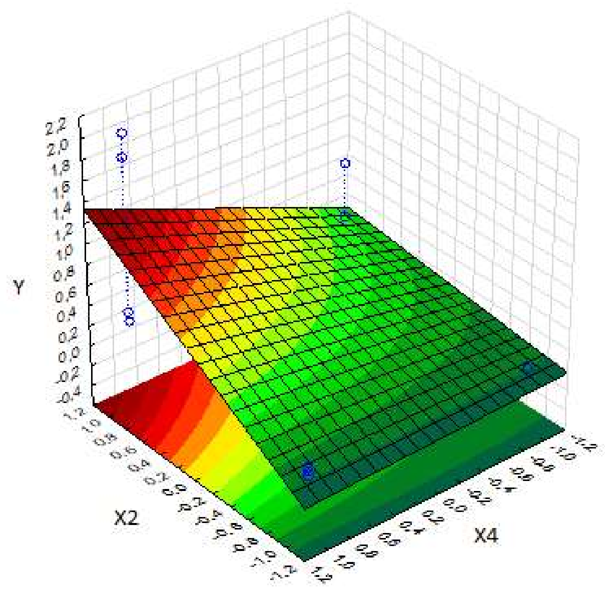

Figure S4 Response Surface of Factorial Design between Variable X2 vs X4

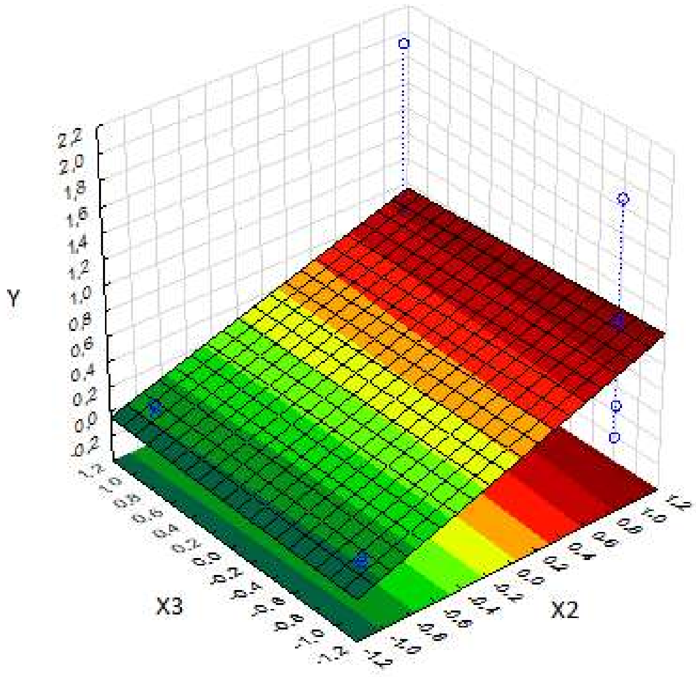

Figure S5 Response Surface of Factorial Design between Variable X2 vs X3

Table S1 - Experiments matrix of 2^4 fractional factorial.

Experiments	(X1)[a]	(X2)[b]	(X3)[c]	(X4)[d]
**1**	-1 (1/63)	-1 (4.0)	-1 (30)	-1 (25)
**2**	-1 (1/63)	1 (8.0)	-1 (30)	-1 (25)
**3**	1	-1 (4.0)	-1 (30)	-1 (25)
**4**	1(1/313)	1 (8.0)	-1 (30)	-1 (25)
**5**	-1(1/63)	-1 (4.0)	1 (180)	-1 (25)
**6**	-1(1/63)	1 (8.0)	1 (180)	-1 (25)
**7**	1(1/313)	-1 (4.0)	1 (180)	-1 (25)
**8**	1(1/313)	1 (8.0)	1 (180)	-1 (25)
**9**	-1(1/63)	-1 (4.0)	-1 (30)	1 (60)
**10**	-1(1/63)	1 (8.0)	-1(30)	1 (60)
**11**	1	-1 (4.0)	-1 (30)	1 (60)
**12**	1(1/313)	1 (8.0)	-1 (30)	1 (60)
**13**	-1(1/63)	-1 (4.0)	1(180)	1 (60)
**14**	-1(1/63)	1 (8.0)	1 (180)	1 (60)
**15**	1(1/313)	-1 (4.0)	1 (180)	1 (60)
**16**	1(1/313)	-1 (8.0)	1 (180)	1 (60)

a X1: enzyme/NPs ratio. b X2: pH. c X3: time (minutes). d X4: temperature (°C).

Table S2 - ANOVA from each independent variable of the factorial. Design 2^4 evaluated at 95% of the coefficient limit.

ANOVA; Var.:Var5; R-sqr=0.94646; Adj:0.83937

Factor	SS	df	MS	F	p
X1	4.57285	1	4.572854	28.46786	0.003101
X2	3.00985	1	3.009845	18.73750	0.007509
X3	0.00466	1	0.004659	0.02900	0.871449
X4	1.60302	1	1.603016	9.97942	0.025123
X1 by X2	2.88367	1	2.883671	17.95202	0.008193
X1 by X3	0.01103	1	0.011027	0.06865	0.803770
X1 by X4	1.38496	1	1.384965	8.62197	0.032395
X2 by X3	0.01334	1	0.013342	0.08306	0.784755
X2 by X4	0.59801	1	0.598012	3.72287	0.111565
X3 by X4	0.11545	1	0.115449	0.71872	0.435259
Error	0.80316	5	0.160632		
Total SS	15.0000	15			

Table S3 - Effect estimates from each independent variable of the Factorial. Design 2^4 evaluated at 95% of the coefficient limit.

Effect Estimates							
Factor	Effect	Std.Err.	t(5)	p	-95% Cnf.Limt	+95% Cnf.Limt	Coeff.
Mean/Interc.	-0.000000	0.100197	-0.00000	1.000000	-0.25757	0.257565	-0.000000
X1	1.069212	0.200395	5.33553	0.003101	0.55408	1.584343	0.534606
X2	-0.867445	0.200395	-4.32868	0.007509	-1.38258	-0.352314	-0.433723
X3	-0.034128	0.200395	-0.17030	0.871449	-0.54926	0.481003	-0.017064
X4	0.633051	0.200395	3.15902	0.025123	0.11792	1.148182	0.316526
X1 by X2	-0.849069	0.200395	-4.23698	0.008193	-1.36420	-0.333938	-0.424534
X1 by X3	-0.052504	0.200395	-0.26200	0.803770	-0.56764	0.462627	-0.026252
X1 by X4	0.588423	0.200395	2.93632	0.032395	0.07329	1.103554	0.294211
X2 by X3	0.057755	0.200395	0.28820	0.784755	-0.45738	0.572886	0.028877
X2 by X4	-0.386656	0.200395	-1.92947	0.111565	-0.90179	0.128475	-0.193328
X3 by X4	0.169889	0.200395	0.84777	0.435259	-0.34524	0.685020	0.084944
